# Effects of Three Different Combined Training Interventions on Jump, Change of Direction, Power Performance, and Inter-Limb Asymmetry in Male Youth Soccer Players

**DOI:** 10.3390/sports9120158

**Published:** 2021-11-24

**Authors:** Alejandro Moreno-Azze, José Luis Arjol-Serrano, David Falcón-Miguel, Chris Bishop, Oliver Gonzalo-Skok

**Affiliations:** 1 Faculty of Health Sciences, University of San Jorge, 50830 Zaragoza, Spain; jlarjol@usj.es; 2 Faculty of Health & Sport Sciences, University of Zaragoza, 50009 Zaragoza, Spain; dfalcon@unizar.es; 3 Faculty of Science and Technology, London Sport Institute, Middlesex University, London NW4 4BT, UK; C.Bishop@mdx.ac.uk; 4 Return to Play Department, Sevilla FC, 41005 Seville, Spain; oligons@hotmail.com

**Keywords:** between-limbs asymmetry, injury prevention, resistance training

## Abstract

Background: This study compared the effects of performing different unilateral combined training interventions on diverse vertical and horizontal jumping performance parameters, change of direction, concentric and eccentric mean power, and their associated inter-limb asymmetries in young soccer players. Methods: Forty-seven young male soccer players (age: 15.5 ± 0.9 years) were distributed into three groups. Two groups performed the same training volume with both legs, beginning with the weaker leg (Stronger Volume Weaker leg group (SVW), *n* = 14) or with the stronger leg (Stronger Volume Stronger leg group, (SVS), *n* = 15). The third group executed double the volume with the weaker leg and also commenced with such leg (Double Volume Weaker leg group (DVW), *n* = 16) during a 10-week period. Pre- and post-intervention tests included a single-leg hop, single-leg lateral hop, triple hop, bilateral and unilateral countermovement jumps, a change of direction speed test, concentric and eccentric mean power during the lateral squat test, and their corresponding asymmetries. Results: Single-leg hop weaker leg, triple hop weaker leg, and bilateral countermovement jump improvements were achieved in the SVW (ES: 0.29 to 0.46) and DVW (ES: 0.55 to 0.73) groups. Between-groups analysis showed better results in single-leg hop in the SVW and DVW compared to group SVS. The DVW group achieved better improvements in countermovement jump in comparison to groups SVS and SVW. Conclusions: Groups that started with the weaker leg seemed to achieve a greater volume of significant changes than when starting with the stronger leg. Performing a double volume on the weaker limb does not guarantee further improved performance compared to other groups.

## 1. Introduction

Sprints, accelerations, decelerations, jumps, and kicks are some of the skills performed during multidirectional team sports and often occur in multiple planes of motion [1,2]. The execution of these skills also often occur in differing volumes between limbs, resulting in the development of asymmetries between the limbs [3].Inter-limb asymmetries are defined as the distinction in execution or capacity of one leg comparative with the other [3], and recent literature has investigated this phenomenon across multiple physical qualities, such as change of direction (COD) speed, jumping, and strength [4,5].

Previous research has suggested that asymmetries > 15% may have higher possibility of lower extremity injury in comparison with scores beneath this cutoff [6]. In conflict with this suggestion, asymmetries that are <10% have been suggested as an acceptable marker to aim for when athletes return to play [7,8] as well as might serve as a protective mechanism against certain injury risk [9,10]. There appears to be no clear consensus regarding the association between inter-limb asymmetry and measures of athletic performance or regarding injury risk and return to play [11,12]. Previous studies have reported significant associations between reduced acceleration or COD speed and unilateral countermovement or drop-jump asymmetries in youth elite team-sports athletes (r = 0.26) and youth female soccer players (r = 0.49–0.59) [13,14]. On the other hand, some studies have stated no meaningful relationships amongst various linear and COD speed tasks and jumping asymmetries [5,15]. In spite of the conflicting evidence, athletes who compete in sports where unilateral movement competency is required (e.g., in team sports) may still be interested in promoting some level of symmetry by encouraging the weaker limb to improve its capacity [16].

To date, there are a variety of training methods that have been employed with the intention to determine the effects on inter-limb asymmetries, such as strength training [17], balance training [18], core stability training [19], eccentric overload training [20], and isoinertial and cable-resistance training [21]. Despite the available evidence, there are only a few studies that have analysed the effects of training on inter-limb asymmetry after a combined resistance training programme [4,22,23]. In spite of this, considerations surrounding training volume or the leg used to begin a given training programme still have not been fully considered. This seems especially relevant given that recent suggestions by Maloney [16] have outlined the importance of improving capacity in the weaker limb.

A previous study with youth male soccer players compared the effects of performing different unilateral eccentric overload training interventions on unilateral and bilateral jumping performance and their related asymmetries. This study showed a substantial enhancement in unilateral jumping in those groups that started the training session with the weaker leg (ES: 0.31 to 0.82) and a significant reduction in between-limbs asymmetries in the triple hop when performing the double volume with the weaker leg (ES: 0.88) [20]. However, to the authors’ knowledge, this is the only study to address which leg to start training with and determine its effects on inter-limb asymmetry.

Therefore, the aim of the current study was to compare the effects of performing different unilateral combined training interventions on change of direction, concentric and eccentric mean power, diverse horizontal and vertical jumping performance parameters, and their related asymmetries in young soccer players. A hypothesis was put forward by the authors that starting the training plan with the weaker leg and performing twice the volume with such leg would be more efficient than starting with the stronger leg or carrying out the same volume on both legs.

## 2. Materials and Methods

### 2.1. Participants

Forty-seven young male (U-17) soccer players (age: 15.5 ± 0.9 years, height: 173.7 ± 7.6 cm, body mass: 64.7 ± 8.2 kg) participated in the study voluntarily. These soccer players belonged to a professional soccer club academy from the Spanish second division. It was assessed that 44 participants were needed in order for the study to have a statistical power of 0.90 with an alpha level of 0.05 and an effect size of 0.5 [24] for a paired *t*-test, using G*Power 3.1 software (University of Düsseldorf, Düsseldorf, Germany). All data were collected among the sixth and eighth month of the season. These eight months were divided into 2 pre-season month periods and 6 competitive month periods. Players had strength/power (1 session) and combined soccer (4 sessions) accumulating ~9 h of programmed training. One competitive match per week was included in the programme. Athletes had an average strength and power training mean experience of 1.89 ± 0.87 years (range: 1 to 4 years). Before the investigation began, players and their legal guardians provided written informed consent. The institutional research ethics committee approved the present study, according to the Declaration of Helsinki recommendations.

### 2.2. Experimental Approach to the Problem

**Figure 1:**
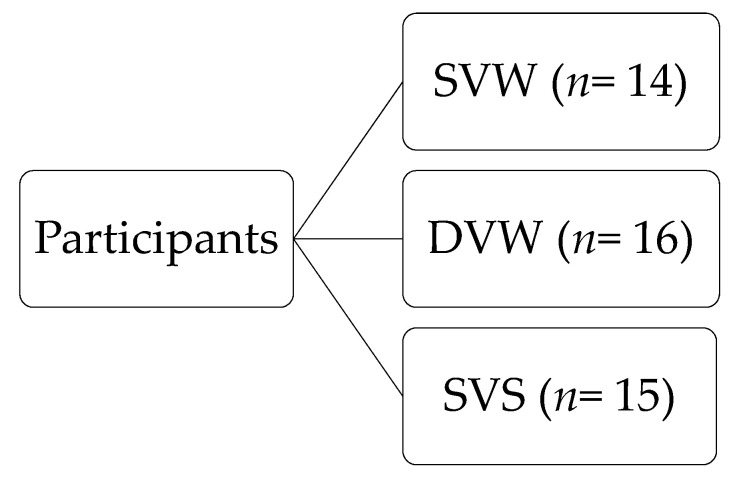
Participants. SVW, group that started all sets with the weaker leg, training the same volume with both legs; DVW, group that began all sets with the weaker leg, training a double volume of sets with the weaker leg in three exercises; SVS, group that started all sets with the stronger leg, training the same volume with both legs.

Based on their initial physical performance ranking, participants were distributed into three unilateral combined training groups (Figure 1), using a controlled and randomized study design (A-B-C-C-B-A distribution). One group started all sets with the weaker leg, training the same volume with both legs (SVW = 14). Another group began with the weaker leg but performed a double volume of sets with the weaker leg in three exercises (DVW = 16). The last group implemented the same volume, beginning with the stronger leg (SVS = 15). The leg that performed better in the majority of jump tests was determined as the strongest leg. For the reliability analysis, tests were performed two weeks before the training period. Testing was conducted one week prior and one week after the training intervention period, with all players familiarized with the exercise technique before the testing period began. The performance of the tests were divided into 2 days per week. On the first day, the functional tests were performed, while the strength test was executed on the second day of the week. A period of 48 h was established between the day of the strength test and the day of the functional test, not performing intense exercise on the day before and consuming their last meal at least 3 h before the scheduled test time was required.

### 2.3. Procedures

#### 2.3.1. Strength Performance Test

The strength performance test was executed the second day of the previous week before the training intervention. Before the session, a standardized warm-up (i.e., 5 min jogging, dynamic stretching, 10 bilateral squats, core exercises, 10 unilateral squats) was performed. This test consisted of 2 sets of lateral squats, beginning with the left leg. A portable conical pulley (VersaPulley, Costa Mesa, CA, USA; inertia 0.27 kg/m^2^, speed:force ratio (i.e., as the ratio increases, the training intensity also increases) 1 out of 4 [25]), and transmission pulley/harness setup from the vest worn was used for this test.

##### Lateral Squat Test

Players were encouraged to perform 10 repetitions. If there was a double warning of the 10% decrement of the first three repetitions mean power before finishing them, they had to stop as well. The best result of two sets from stronger (S) and weaker (W) leg were used for further analysis, leaving 1 and 3 min of recovery between legs and sets, respectively. A specific software (SmartCoach ® v.5.6.0.8, SmartCoach Europe AB, Stockholm, Sweden) recorded mean concentric (ConMean) and eccentric (EccMean) power. Thus, mean concentric power stronger leg (ConMean stronger), mean concentric power weaker leg (ConMean weaker), mean eccentric power stronger leg (EccMean stronger), and mean eccentric power weaker leg (EccMean weaker) were the final variables analysed.

#### 2.3.2. Functional Performance Tests

The first day of the previous week before the training intervention, tests were performed after a 10-min standardized warm-up (i.e., 5-min jogging, dynamic stretching, 10 bilateral squats, 10 unilateral squats, 3 vertical unilateral jumps, and core exercises). Participants were required to start with the left leg. Single-leg hop (SLH), triple hop (TH), bilateral and unilateral countermovement jump (CMJ), single-leg lateral hop (SLLH), and 180° change of direction (COD) was the order of these tests.

##### Single-Leg Hop Test

Standing on the test leg with both hands held behind the lower back, players were encouraged to hop as far as they could, landing with the same leg. For this, swinging the free leg was allowed at the push-off. A controlled balanced landing was required, keeping the landing foot in place for at least 2–3 s (any extra hop or slip were not considered valid). Recovery was 30 s between jumps and 2 min between legs. The distance from the toe at the beginning to the heel when the athlete landed was assessed in centimetres. The best of three jumps with each leg was measured, acquiring results in the stronger (SLH stronger) and weaker (SLH weaker) legs for further analysis. 

##### Single-Leg Lateral Hop Test

Standing sidewise on the test leg with both hands held behind the lower back, players were encouraged to hop as far as they could, landing with the same leg in the same position. For this, swinging the free leg was allowed at the push-off. Landing with the other leg was allowed, as long as the tested leg landed first. Recovery was 30 s between jumps and 2 min between legs. The distance from the toe at the beginning to the nearest part of the foot to the push-off point when the athlete landed was assessed in centimetres. The best of three jumps with each leg was measured, acquiring results in the stronger (SLLH stronger) and weaker (SLLH weaker) legs for further analysis.

##### Triple Hop Test

Standing on the test leg with both hands held behind the lower back, players were encouraged to hop three times with the same leg as far as they could. For this, swinging the free leg was allowed at the push-off. A controlled balanced landing was required, keeping the landing foot in place for at least 2–3 s (any extra hop or slip were not considered valid). Recovery was 30 s between jumps and 2 min between legs. The distance from the toe at the beginning to the heel when the athlete landed was assessed in centimetres. The best of three jumps with each leg was measured, acquiring results in the stronger (TH stronger) and weaker (TH weaker) legs for further analysis. 

##### Bilateral CMJ Test

Jump height, in centimetres, was assessed calculating the flight time using an Optojump (Microgate, Bolzano, Italy), which had been validated against a force platform. Standing with both hands on their hips during the test, players had 3 attempts, selecting the best jump for future analysis. Players had 30 s of passive recovery between jumps, and the depth of the CMJ was not standardized, allowing participants to decide the depth of the CMJ. 

##### Unilateral CMJ Test

Standing on the test leg with both hands on their hips during the test, players were encouraged to jump as high as possible (Optojump, Microgate, Bolzano, Italy). For this, swinging the free leg was allowed at the push-off, as long as it was flexed to 90° at the hip and knee. A controlled balanced landing was required, keeping the landing foot in place for at least 2–3 s (any extra hop or slip were not considered valid). Recovery was 30 s between jumps and 2 min between legs. The distance from the toe at the beginning to the heel when the athlete landed was assessed in centimetres. The best of three jumps with each leg was measured, acquiring results in bilateral (CMJ), the stronger (CMJ stronger), and weaker (CMJ weaker) legs for further analysis.

##### Change of Direction Speed Test

Change of direction speed test (COD) consisted of 5 m sprinting in a straight line and turning 180° back to the starting point. Players placed the front foot 0.5 m before the first gate, which carried photocells (Witty, Microgate, Bolzano, Italy) that recorded the time. Players had 2 attempts with each leg, and the fastest time was used for analysis, acquiring the stronger leg (COD stronger) and weaker leg (COD weaker). Between attempts, a 60-s recovery period took place. To consider any attempt properly done, the entire foot had to pass all the line in the change of direction.

#### 2.3.3. Training Intervention

**Figure 2:**
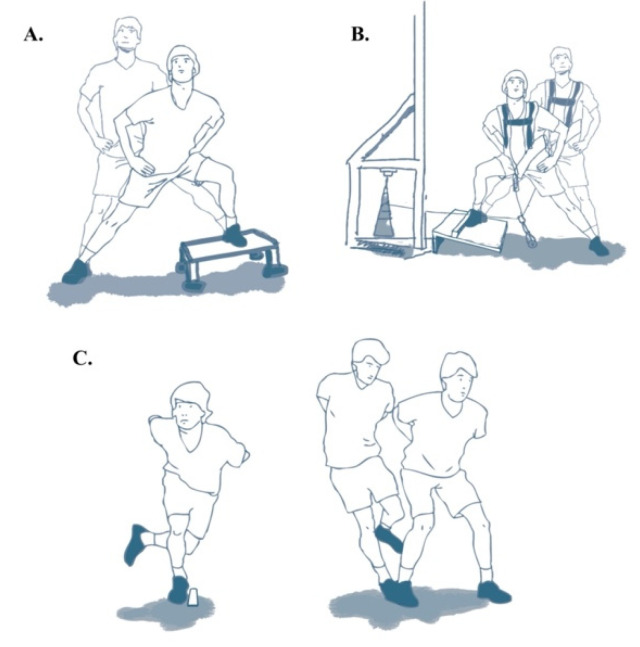
Unilateral combined training programme. (**A**) Lateral squat on a custom-made vibration platform, (**B**) lateral squat in a Versapulley, and (**C**) single-legged lateral hop.

The performance of one unilateral combined training session per week was added to the participants on their normal soccer training programme. These sessions (Table 1) were developed in the respective 48 h after the previous match and 48-h prior to the subsequent match. This training intervention consisted in a lateral squat on a custom-made vibration platform (30-Hz frequency; custom-made platform, Laboratory of Human Performance, VFSport, Seville, Spain), lateral squat using a Versapulley (Costa Mesa, CA, USA; inertia 0.27 kg/m^2^, speed: force ratio (i.e., as the ratio increases, the training intensity also increases) 1–3 out of 4 [25]), and single-legged side horizontal jump (Figure 2). Before the training intervention (Table 1), participants did a standardized warm up (i.e., 5 min jogging, dynamic stretches and 2 sets of lateral squats with each leg of 8 repetitions doing the last 3 repetitions as fast as possible). The exclusion criteria for the analysis was established for those players who did not complete at least 80% of the training sessions. Strength performance out of the sagittal plane was suggested because soccer players frequently perform multidirectional movement patterns, and many mechanisms of injury often occur in the frontal plane [26]. All training sessions were controlled by the main researcher and three experienced strength and conditioning coaches who provided verbal encouragement to each participant.

### 2.4. Statistical Analyses

All data are presented as mean ± standard deviation (SD). The Shapiro–Wilk test was used to analyze the normally distributed data. The paired *t*-test was applied to detect significant differences for within group comparisons, established a priori at *p* < 0.05 in any variable. All data were log-transformed before analysing in order to avoid any bias from a non-uniformity error. Between-session reliability analysis was examined by a two-way random intraclass correlation coefficient (ICC) with absolute agreement and 90% confidence interval in addition to the CV. The CV values were considered acceptable if <10% [27], while ICC values > 0.9 = excellent, 0.75–0.9 = good, 0.5–0.75 = moderate, and <0.5 = poor [28].To determine the effect size (ES, 90% CI), pooled pre-training SD in the selective variables was used. Cohen’s d ES statistics threshold values were >0.2 (small), >0.6 (moderate), and >1.2 (large) [29]. The differences in performance chances for within/between-group comparisons were calculated, being better/greater, similar, or worse/smaller. Quantitative chances of beneficial/better or detrimental/poorer effect were assessed qualitatively as follows: <1%, most likely not; >1–5%, very unlikely; >5–25%, unlikely; >25–75%, possible; >75–95%, likely; >95–99%, very likely; and >99%, most likely likely [29]. If the chance that the true value is >25% beneficial and >0.5% chance that it is harmful, the clinical effect was considered as unclear.

If the odds ratio of benefit/harm was <66, it continued being unclear. When odds ratio of benefit/harm was >66, the clinical inference was declared as beneficial. For between-group (xCompare2groups.xls) and within-group (xPostOnlyCrossover.xls) analysis, two specific Excel spreadsheets from sportsci.org (https://sportsci.org/index.html) were used.

Inter-limb asymmetries were calculated using this formula [30]:

100/Max Value (right and left) × Min Value (right and left) x − 1 + 100 (1)

## 3. Results

### 3.1. Participants

Because of changes in their training plan or not completing 80% of the training sessions, two participants were excluded. Therefore, 45 participants (15.5 ± 0.9 years, 173.4 ± 7.7 cm, 64.6 ± 8.3 kg) were analysed. During the unilateral combined training sessions, no injuries were registered. Finally, this resulted in 14 participants in the SVW, 16 for DVW, and 15 for SVS groups. Once these dropouts had been considered, no significant differences were found between groups at baseline.

### 3.2. Reliability Analysis

When considering the CV, the strength test showed poor reliability, with all measures exhibiting values >10%. Functional performance tests showed acceptable values with all being <10%, with the exception of the CMJ on the weaker leg, which showed a CV of 13.14%. When considering ICC data, all tests showed moderate to good reliability (Table 2).

### 3.3. Within-Group Changes

Group SVW obtained likely improvements in SLH stronger, SLH weaker, TH weaker, CMJ, CMJ stronger, COD weaker, ConMean weaker, EccMean stronger, and EccMean weaker; very likely enhancements in SLLH stronger, SLLH weaker, and ConMean stronger; and most likely changes in TH stronger. The DVW group achieved likely enhancements in SLH weaker, COD weaker, ConMean stronger, and EccMean stronger; very likely outcomes in SLLH stronger and CMJ; and most likely improvements in SLLH weaker, TH stronger, TH weaker, CMJ stronger, and CMJ weaker. The SVS group reached likely improvements TH stronger and CMJ stronger; very likely effects in SLLH weaker, ConMean stronger, and ConMean weaker; and most likely results in EccMean stronger and EccMean weaker.

### 3.4. Between-Group Changes

**Figure 3:**
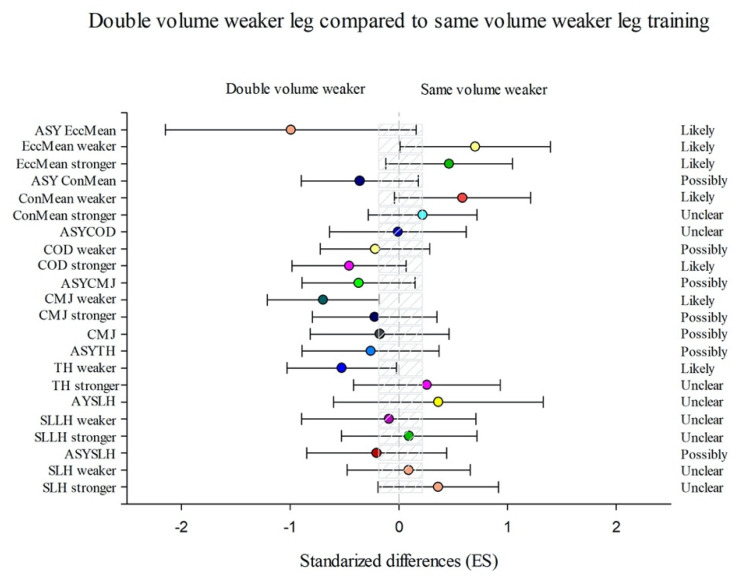
Efficiency of the unilateral combined training performing the double volume with the weaker leg starting with the weaker leg (DVW) compared with the unilateral combined training performing the same volume with both legs starting with the weaker leg (SVW) training programme to improve a single-leg hop (SLH) with the stronger and the weaker leg and the corresponding asymmetry (AsySLH), single-leg lateral hop (SLLH) with the stronger and the weaker leg and the corresponding asymmetry (AsySLLH), triple hop (TH) with the stronger and the weaker leg and the corresponding asymmetry (AsyTH), bilateral countermovement jump (CMJ), single-leg countermovement jump (CMJ) with the stronger and the weaker leg and the corresponding asymmetry (AsyCMJ), change of direction (COD) with the stronger and the weaker leg and the corresponding asymmetry (AsyCOD), lateral squat in mean concentric power (ConMean) with the stronger and the weaker leg and the corresponding asymmetry (AsyConMean), and mean eccentric power (MeanEcc) with the stronger and the weaker leg and the corresponding asymmetry (AsyEccMean) (Bars indicate uncertainty in the true mean changes with 90% confidence limits.) Trivial areas were the smallest worthwhile change (see Section 2).

**Figure 4:**
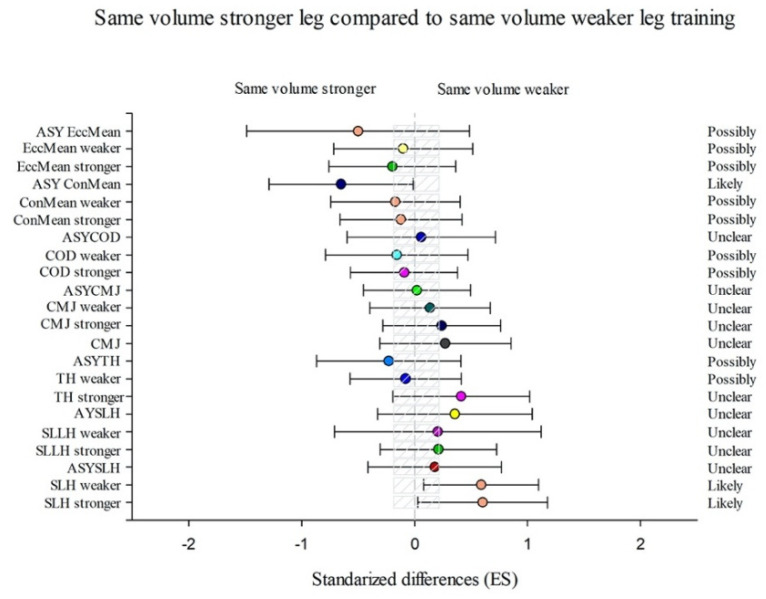
Efficiency of the unilateral combined training performing the same volume with both leg starting with the stronger leg (SVS) compared with the unilateral combined training performing the same volume with both legs starting with the weaker leg (SVW) training programme to improve a single-leg hop (SLH) with the stronger and the weaker leg and the corresponding asymmetry (AsySLH), single-leg lateral hop (SLLH) with the stronger and the weaker leg and the corresponding asymmetry (AsySLLH), triple hop (TH) with the stronger and the weaker leg and the corresponding asymmetry (AsyTH), bilateral countermovement jump (CMJ), single-leg countermovement jump (CMJ) with the stronger and the weaker leg and the corresponding asymmetry (AsyCMJ), change of direction (COD) with the stronger and the weaker leg and the corresponding asymmetry (AsyCOD), an lateral squat in mean concentric power (ConMean) with the stronger and the weaker leg and the corresponding asymmetry (AsyConMean), and mean eccentric power (MeanEcc) with the stronger and the weaker leg and the corresponding asymmetry (AsyEccMean) (Bars indicate uncertainty in the true mean changes with 90% confidence limits.) Trivial areas were the smallest worthwhile change (see Section 2).

The SVW group showed more substantial enhancements in ConMean weaker (15.84% (CL90%−1.04; 35.59))), EccMean stronger (11.92% (CL90%−2.92; 29.02)), and EccMean weaker (19.36% (CL90%0.24; 42.12)) than DVW (Figure 3) and considerable enhancements in SLH stronger (4.05% (CL90%0.19; 8.06)) and SLH weaker (4.81% (CL90%0.66; 9.13)) than the SVS group (Figure 4).

**Figure 5:**
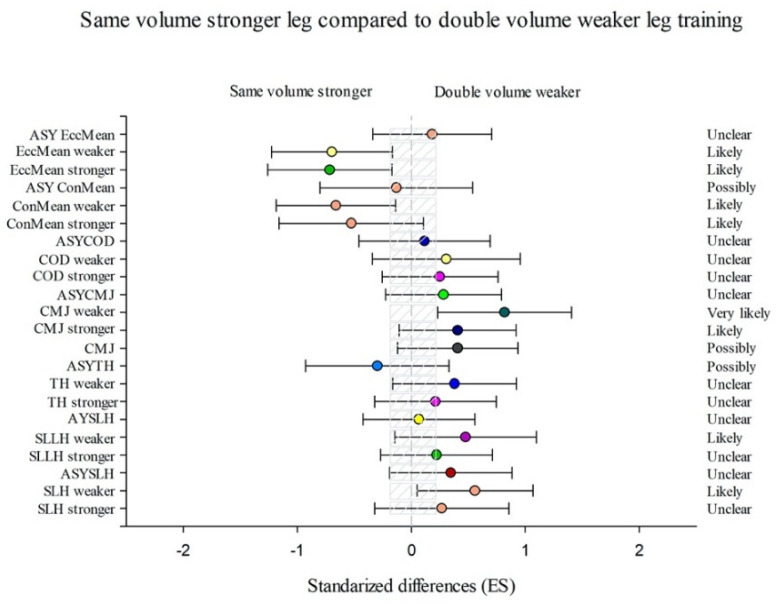
Efficiency of the unilateral combined training performing the same volume with both legs starting with the stronger leg (SVS) compared with the unilateral combined training performing the double volume with the weaker leg starting with the weaker leg (DVW) training programme to improve a single-leg hop (SLH) with the stronger and the weaker leg and the corresponding asymmetry (AsySLH), single-leg lateral hop (SLLH) with the stronger and the weaker leg and the corresponding asymmetry (AsySLLH), triple hop (TH) with the stronger and the weaker leg and the corresponding asymmetry (AsyTH), bilateral countermovement jump (CMJ), single-leg countermovement jump (CMJ) with the stronger and the weaker leg and the corresponding asymmetry (AsyCMJ), change of direction (COD) with the stronger and the weaker leg and the corresponding asymmetry (AsyCOD), an lateral squat in mean concentric power (ConMean) with the stronger and the weaker leg and the corresponding asymmetry (AsyConMean), and mean eccentric power (MeanEcc) with the stronger and the weaker leg and the corresponding asymmetry (AsyEccMean) (Bars indicate uncertainty in the true mean changes with 90% confidence limits.) Trivial areas were the smallest worthwhile change (see Section 2).

Group SVS showed more substantial training effects in TH weaker (−0.42% (CL90%−2.96; 2.18)), COD stronger (−0.4% (CL90%−2.42; 1.66)), and ASYConMean (−54.02% (CL90%−78.59; −1.27)) than SVW group (Figure 4) and better results in ConMean stronger (−11.15% (CL90%−22.92; 2.41)), ConMean weaker (−19.22% (CL90%−31.81; −4.31)), EccMean stronger (−18.29% (CL90%−29.92; −4.73)), and EccMean weaker (−20.28% (CL90%−32.89; −5.3)) than DVW group (Figure 5).

The DVW group showed more substantial differences in TH weaker (−2.35% (CL90%−4.55; −0.1)), CMJ stronger (−2.43% (CL90%−8.39; 3.92)), CMJ weaker (−9.75% (CL90%−16.32; −2.65)), and ASYEccMean (−59.78% (CL90% −86.03; 15.78)) than SVW group (Figure 3) and considerable enhancements in SLH stronger (1.78% (CL90%−2.1; 5.82)), SLH weaker (4.56% (CL90%0.4; 8.89)), SLLH stronger (1.79% (CL90%−2.14; 5.87)), TH stronger (0.89% (CL90%−1.34; 3.17)),CMJ stronger (6.26% (CL90%−1.58; 14.72)), CMJ weaker (12.59% (CL90%3.43; 22.56)), and COD stronger (0.98% (CL90%−0.99; 3)) than SVS group (Figure 5).

## 4. Discussion

The present study compared the effects of performing different unilateral combined training interventions on various measures of athletic performance in male youth soccer players. The main findings of the study were: (1) likely training effects in SLH were found in SVW group with the stronger and weaker leg; (2) likely improvements were found in SLH weaker leg in DVW group; (3) most and very likely improvements were found in SLLH and TH with both stronger and weaker leg in those groups that started with the weaker leg; (4) substantial enhancement in CMJ bilateral and with both stronger and weaker leg in those groups that started the training programme with the weaker leg; (5) likely improvements in COD weaker were reached in those groups that performed the same volume in both legs; and (6) moderate and likely asymmetry reduction were achieved in TH and COD, respectively, in the DVW group.

The first point to consider from these results is that starting with the weaker leg seems like a strong consideration for bringing about a greater number of significant changes than when starting with the stronger leg (Table 3). Table 3 shows the changes in performance and asymmetries and indicates significant improvements in 24 tests when starting with the weaker leg (13 tests in SVW group and 11 tests in DVW group), whilst only seven significant improvements when starting with the stronger leg (SVS group). These findings are in agreement with a previous study with male youth soccer players, which compared the effects of performing different unilateral strength training interventions on unilateral and bilateral jumping performance and their related asymmetries. In this study, substantial enhancements were achieved in TH stronger (ES: 0.51 to 0.53), TH weaker (ES: 0.71 to 0.79), and CMJ weaker (ES: 0.31 to 0.68) in those groups that began their training programme with the weaker leg in comparison with the group that started this intervention with the stronger leg (ES: 0.00 in TH stronger, 0.15 in TH weaker, and 0.23 in CMJ weaker) [20]. This is the second study to show potentially greater benefits from training with the weaker limb first. Although anecdotal, it is possible that by prioritizing the weaker limb first, especially in an exercise that is not truly unilateral (as per the present study), it is less impacted by any fatigue during training and, over time, enables athletes to maximize their physical adaptation from the intervention.

Despite starting with the weaker showing a greater volume of significant changes than when starting with the stronger leg, this does not always guarantee the weaker leg gets more benefit out of the two limbs. The SVW group achieved substantial improvements in CMJ stronger (ES: 0.55) and COD weaker (ES: 0.50), while no significant changes were obtained in CMJ weaker (ES: 0.15) and COD stronger (ES: 0.30). This also happened in group DVW, which showed substantial improvements in SLH weaker (ES: 0.55), COD weaker (ES: 0.45), ConMean stronger (ES: 0.36), and EccMean stronger (ES: 0.32), while no significant changes were achieved in SLH stronger (ES: 0.01), COD stronger (ES: 0.21), ConMean weaker (ES: 0.17), and EccMean weaker (ES: 0.26).

Another important finding of the present study is that doing double the volume on the weaker side does not guarantee further improved performance compared to the other two groups. Looking at Table 3, the DVW group only showed better results in six tests in comparison with groups SVW and SVS. In the rest of the tests, group DVW obtained lower significant changes in comparison with the SVS and SVW groups or no substantial improvements. Previous research has highlighted that unilateral tests produce much greater variability than bilateral, not guaranteeing consistent improvements in their results [31,32]. Thus, these findings could have been influenced because many of the selected outcome measures were performed unilaterally. Therefore, bilateral and unilateral tests differences involving the performance of different volume in each leg during several combined training programmes in further studies are warranted.

Previous research has suggested that unilateral training may help reduce between-limb asymmetries [4,18], supporting the significance of asymmetries for both protection against injuries and athletic performance [4,11]. Recent studies suggested that asymmetries are both test- and metric-specific due to their inherent variability during testing; thus, the individual nature of asymmetry must be acknowledged here. In the present study, a lower range of asymmetry reduction (ES: −0.71 to 0.30) was acquired in comparison to other research studies (ES: −0.62 to 1.15) [4,22,23]. It should be acknowledged that the exercise selected for this particular intervention is not considered totally unilateral. Thus, and given our previous suggestion relating to the efficacy of unilateral training for the reduction of asymmetry, it is possible that other (more unilateral) exercises would have greater or lesser benefit (e.g., single leg squats, step ups, etc.). Another interesting finding in the current study is the moderate asymmetry reduction that the DVW group achieved in TH (ES: 0.30) in comparison with the groups that performed the same volume in both legs in their training programme (ES: −0.52 for the SVW group and −0.71 for group SVS). No substantial differences were found in training volume or the leg used to start the intervention employing the same exercise in between-groups differences. These findings could be in accordance with a previous study, which showed extreme differences between the individual asymmetry scores and the mean values for all metrics, highlighting the necessity for a more individualized approach to asymmetry analysis [33].

Nevertheless, some limitations are acknowledged in the current study. Firstly, in order to determine true cause and effect, the inclusion of a control group would have been useful in this study. However, given the present sample were all used from one club, it is virtually impossible to request that some players do not conduct any training. As such, this often serves as a pitfall in research in elite sport settings. Second, the design of an alternative training intervention where the chosen exercise(s) are purely unilateral (e.g., single leg squat, step up) could be an interesting line of investigation for future research. In turn, assessing power using isokinetic devices could be interesting for future studies. Finally, future research comparing different combined training effects on performance and their inter-limbs asymmetries in other athletic samples with different age, gender, and sport are needed.

## 5. Conclusions

Likely training effects in SLH were found in SVW group with the stronger and weaker leg. Likely improvements were found in SLH weaker leg in DVW group, most and very likely improvements were found in SLLH and TH with both stronger and weaker leg in those groups that started with the weaker leg. Substantial enhancement was found in CMJ bilateral and with both stronger and weaker leg in those groups that started the training programme with the weaker leg. Likely improvements in COD weaker were reached in those groups that performed the same volume in both legs. Moderate and likely asymmetry reduction were achieved in TH and COD, respectively, in the DVW group. Attending to the principle of specificity in team sports, which involves applying one-sided force, unilateral training should be included. Moreover, the present study highlights that if practitioners start their strength training sessions with the weaker limb first (which of course must be pre-determined), it may carry over to greater improvements in overall athletic performance.

## Figures and Tables

**Table 1:** Unilateral Combined training programme.

	Lateral Squat on Vibration-Platform			Lateral Squat			Unilateral Side Hop	
	Sets/Leg	Repetitions	Hz	Sets/Leg	Repetitions	Speed/Force Ratio	Sets/Leg	Repetitions
Session 1	1	6	30	2	6	1 out of 4	2	4
Session 2	1	6	30	2	6	1 out of 4	2	4
Session 3	1	8	30	2	8	1 out of 4	2	5
Session 4	1	8	30	2	8	1 out of 4	2	5
Session 5	1	8	30	2	8	2 out of 4	2	5
Session 6	1	8	30	2	8	2 out of 4	2	5
Session 7	1	10	30	2	10	2 out of 4	2	6
Session 8	1	10	30	2	10	2 out of 4	2	6
Session 9	1	10	30	2	10	3 out of 4	2	6
Session 10	1	10	30	2	10	3 out of 4	2	6

**Table 2:** Measures of pre-intervention reliability in strength and functional performance tests (*n* = 47).

TEST	Difference (90% CL)	TEM (90% CL)	CV (90% CL)	ICC (90% CL)
SLH stronger (cm)	−1.71 (−3.79; 0.37)	5.88 (5.02; 7.15)	3.53 (3.01; 4.31)	0.80 (0.69; 0.87)
SLH weaker (cm)	−1.51 (−3.76; 0.74)	6.36 (5.42; 7.73)	4.02 (3.42; 4.91)	0.77 (0.65; 0.86)
SLLH stronger (cm)	−2.18 (−4.67; 0.32)	7.04 (6.00; 8.55)	5.00 (4.25; 6.11)	0.81 (0.70; 0.88)
SLLH weaker (cm)	−0.49 (−2.75; 1.77)	6.39 (5.45; 7.76)	4.56 (3.87; 5.57)	0.79 (0.68; 0.87)
TH stronger (cm)	−4.70 (−8.97; −0.43)	11.78 (10.01; 14.39)	2.16 (1.83; 2.64)	0.89 (0.82; 0.93)
TH weaker (cm)	−7.53 (−12.70; −2.37)	14.23 (12.09; 17.38)	2.82 (2.39; 3.45)	0.86 (0.78; 0.92)
CMJ (cm)	1.07 (0.41; 1.73)	1.92 (1.64; 2.31)	5.76 (4.92; 7.00)	0.86 (0.79; 0.91)
CMJ stronger (cm)	0.33 (−0.16; 0.82)	1.39 (1.18; 1.69)	7.89 (6.69; 9.67)	0.76 (0.63; 0.84)
CMJ weaker (cm)	0.86 (0.12; 1.59)	2.07 (1.76; 2.51)	13.14 (11.1; 16.19)	0.54 (0.34; 0.69)
COD stronger (s)	0.04 (0.02; 0.06)	0.05 (0.04; 0.06)	1.90 (1.62; 2.31)	0.74 (0.61; 0.83)
COD weaker (s)	0.03 (0.01; 0.05)	0.06 (0.05; 0.07)	2.09 (1.78; 2.55)	0.69 (0.54; 0.80)
ConMean stronger (W)	58.23 (32.42; 84.03)	70.27 (59.63; 86.08)	23.36 (19.50; 29.32)	0.84 (0.74; 0.90)
ConMean weaker (W)	51.14 (19.81; 82.47)	85.31 (72.39; 104.50)	19.91 (16.66; 24.91)	0.85 (0.77; 0.91)
EccMean stronger (W)	58.7 (26.33; 91.06)	88.13 (74.79; 107.96)	31.63 (26.26; 40.02)	0.74 (0.60; 0.84)
EccMean weaker (W)	44.63 (19.73; 69.54)	67.82 (57.55; 83.07)	23.79 (19.85; 29.87)	0.81 (0.70; 0.88)

SLH, single-leg hop with the stronger and the weaker leg; SLLH, single-leg lateral hop with the stronger and the weaker leg; TH, triple hop with the stronger and the weaker leg; CMJ, countermovement jump bilateral and with the stronger and the weaker leg; COD, change of direction with the stronger and the weaker leg; ConMean, mean concentric power output with the stronger and the weaker leg; EccMean, mean eccentric power output with the stronger and the weaker leg; CL, confidence limit; CV, coefficient of variation expressed as percentage of TEM; TEM, typical error of measurement; Difference, difference in mean between the 2 trials; ICC, intraclass correlation coefficient.

**Table 3:** Performance and asymmetries changes after unilateral combined training with different strategies.

	SVW = Same Volume, Weaker Leg (*n* = 14)				DVW = Double Volume, Weaker Leg (*n* = 16)				SVS = Same Volume, Stronger Leg (*n* = 15)			
Variables	Pre-Test	Post-Test	ES (CL90%)	*p*	Pre-Test	Post-Test	ES (CL90%)	*p*	Pre-Test	Post-Test	ES (CL90%)	*p*
SLH stronger (cm)	168.89 ± 11.00	173.79 ± 13.81	0.40 (0.02; 0.78)	<0.01	171.78 ± 12.65	172.06 ± 15.04	0.01 (−0.36; 0.37)	<0.01	171.37 ± 13.56	168.60 ± 11.98	−0.19 (−0.57; 0.20)	<0.01
SLH weaker (cm)	165.04 ± 14.02	171.79 ± 11.14	0.46 (0.13; 0.78)	0.01	165.28 ± 9.93	171.13 ± 11.45	0.55 (0.18; 0.91)	<0.01	168.80 ± 14.51	166.40 ± 14.99	−0.16 (−0.56; 0.23)	<0.01
ASY_SLH_ (%)	3.96 ± 3.33	3.46 ± 2.75	0.06 (−0.53; 0.66)	0.26	3.71 ± 3.24	3.91 ± 2.85	−0.14 (−0.79; 0.50)	<0.01	3.62 ± 2.43	3.31 ± 2.85	−0.05 (−0.81; 0.71)	0.04
SLLH stronger (cm)	142.89 ± 15.24	151.64 ± 9.96	0.55 (0.20; 0.90)	0.01	143.41 ± 14.51	151.63 ± 14.71	0.52 (0.32; 0.73)	<0.01	146.80 ± 15.58	151.80 ± 15.73	0.3 (−0.02; 0.61)	<0.01
SLLH weaker (cm)	137.00 ± 11.90	149.57 ± 13.41	0.90 (0.32; 1.48)	0.57	140.41 ± 11.45	152.25 ± 13.87	0.92 (0.63; 1.21)	<0.01	144.57 ± 13.62	152.40 ± 15.44	0.51 (0.29; 0.73)	<0.01
ASY_SLLH_ (%)	6.95 ± 4.53	6.69 ± 3.75	−0.51 (−1.51; 0.48)	0.10	4.30 ± 3.81	5.43 ± 4.98	−0.04 (−0.54; 0.47)	0.06	5.13 ± 4.31	5.42 ± 4.70	0.02 (−0.67; 0.71)	0.80
TH stronger (cm)	550.89 ± 30.21	577.50 ± 31.4	0.82 (0.48; 1.16)	<0.01	545.97 ± 26.47	568.19 ± 32.94	0.76 (0.50; 1.03)	<0.01	553.13 ± 44.99	569.60 ± 38.44	0.36 (0.11; 0.62)	<0.01
TH weaker (cm)	538.29 ± 38.77	550.00 ± 39.61	0.29 (0.10; 0.47)	<0.01	539.53 ± 30.40	564.19 ± 36.32	0.73 (0.43; 1.04)	<0.01	538.50 ± 47.13	552.73 ± 43.53	0.30 (0.02; 0.57)	<0.01
ASY_TH_ (%)	3.25 ± 2.81	5.64 ± 4.39	−0.52 (−1.04; −0.01)	0.19	2.77 ± 1.55	2.93 ± 3.41	0.30 (−0.22; 0.82)	<0.01	3.55 ± 2.52	4.56 ± 2.53	−0.71 (−1.64; 0.22)	0.08
CMJ (cm)	33.91 ±4.37	35.89 ±4.79	0.42 (0.10; 0.73)	<0.01	32.90 ±3.41	35.51 ±3.84	0.66 (0.33; 0.99)	<0.01	32.51 ±4.50	33.85 ±4.35	0.29 (−0.06; 0.63)	<0.01
CMJ stronger (cm)	19.04 ± 2.33	20.36 ± 1.98	0.55 (0.09; 1.01)	0.15	17.99 ± 2.36	20.45 ± 2.48	0.95 (0.56; 1.33)	<0.01	17.69 ± 2.97	19.19 ± 3.24	0.46 (0.07; 0.85)	<0.01
CMJ weaker (cm)	18.29 ± 2.46	17.26 ± 5.36	0.15 (−0.29; 0.58)	0.04	17.24 ± 2.27	20.14 ± 3.19	1.10 (0.64; 1.55)	<0.01	16.69 ± 3.27	17.60 ± 3.07	0.28 (−0.07; 0.63)	<0.01
ASY_CMJ_ (%)	6.52 ± 3.14	9.58 ± 7.08	−0.15 (−0.70; 0.39)	0.94	8.24 ± 6.92	9.80 ± 4.87	−0.51 (−1.08; 0.07)	0.01	7.70 ± 7.44	10.02 ± 9.12	−0.15 (−0.58; 0.27)	0.11
COD stronger (s)	2.74 ± 0.07	2.71 ± 0.08	0.30 (0.02; 0.58)	<0.01	2.80 ± 0.07	2.78 ± 0.13	0.21 (−0.19; 0.60)	<0.01	2.77 ± 0.12	2.75 ± 0.13	0.15 (−0.23; 0.54)	<0.01
COD weaker (s)	2.75 ± 0.06	2.71 ± 0.08	0.50 (0.04; 0.97)	0.36	2.79 ± 0.10	2.75 ± 0.08	0.45 (−0.07; 0.96)	<0.01	2.74 ± 0.11	2.72 ± 0.10	0.24 (−0.10; 0.57)	<0.01
ASYCOD (%)	2.40 ± 1.70	2.98 ± 1.81	−0.45 (−1.05; 0.15)	0.24	2.60 ± 1.76	2.72 ± 1.97	−0.15 (−0.81; 0.51)	<0.01	2.60 ± 2.16)	1.70 ± 1.98	0.52 (0.03; 1.01)	<0.01
ConMean stronger (W)	545.58 ± 225.01	654.18 ± 211.19	0.46 (0.27; 0.65)	<0.01	471.50 ± 182.00	563.20 ± 239.99	0.36 (0.15; 0.58)	<0.01	478.65 ± 179.92	607.30 ± 157.77	0.68 (0.39; 0.98)	<0.01
ConMean weaker (W)	550.03 ± 218.49	630.23 ± 186.92	0.38 (0.07; 0.69)	0.01	490.34 ± 219.59	513.66 ± 176.68	0.17 (−0.05; 0.38)	<0.01	450.30 ± 181.00	599.56 ± 175.16	0.66 (0.33; 1.00)	<0.01
ASYConMean (%)	9.64 ± 10.30	9.80 ± 6.68	−0.41 (−1.35; 0.53)	0.06	15.36 ± 13.48	13.16 ± 9.34	−0.13 (−0.87; 0.60)	0.90	13.61 ± 11.72	15.19 ± 11.25	−0.28 (−1.12; 0.56)	0.73
EccMean stronger (W)	475.64 ± 210.27	576.63 ± 169.38	0.49 (0.17; 0.80)	<0.01	432.13 ± 177.75	499.42 ± 203.18	0.32 (0.11; 0.52)	<0.01	405.80 ± 156.64	565.01 ± 152.05	0.91 (0.50; 1.32)	<0.01
EccMean weaker (W)	452.16 ± 165.74	575.26 ± 183.53	0.61 (0.18; 1.05)	0.07	435.65 ± 196.39	477.13 ± 168.83	0.26 (0.02; 0.49)	<0.01	378.06 ± 157.76	547.00 ± 152.41	0.86 (0.50; 1.22)	<0.01
ASYEccMean (%)	16.73 ± 18.60	9.56 ± 4.37	−0.65 (−2.30; 1.00)	0.85	18.19 ± 12.09	11.69 ± 7.51	0.46 (−0.25; 1.17)	0.02	16.54 ± 16.58	13.82 ± 12.96	0.18 (−0.29; 0.65)	0.71

SLH, single-leg hop with the stronger and the weaker leg; Asy_SLH_, asymmetry in the single-leg hop; SLLH, single-leg lateral hop with the stronger and the weaker leg; Asy_SLLH_, asymmetry in the single-leg lateral hop; TH, triple hop with the stronger and the weaker leg; Asy_TH_, asymmetry in the triple hop; CMJ, bilateral countermovement jump and with the stronger and the weaker leg; Asy_CMJ_, asymmetry in the unilateral countermovement jump; ConMean, mean concentric power output with the stronger and the weaker leg; ASYConMean, asymmetry in the mean concentric power output; EccMean, mean eccentric power output with the stronger and the weaker leg; ASYEccMean, Asymmetry in the mean eccentric power output; ES, effect size; CL, confidence limit; SVW, unilateral combined training performing the same volume with both limbs starting with the weaker limb; DVW, unilateral combined training performing the double volume with the weaker limb starting with the weaker limb; SVS, unilateral combined training performing the same volume with both limbs starting with the stronger limb. All results are presented in the same direction; that is, a positive change is considered an improvement, while a negative change is considered an impairment.
